# The Synergy between Nuclear Magnetic Resonance and Density Functional Theory Calculations

**DOI:** 10.3390/molecules29020336

**Published:** 2024-01-09

**Authors:** Poul Erik Hansen

**Affiliations:** Department of Science and Environment, Roskilde University, DK-4000 Roskilde, Denmark; poulerik@ruc.dk

**Keywords:** DFT calculations, tautomerism, hydrogen bonding, isotope effects, natural products

## Abstract

This paper deals with the synergy between Nuclear Magnetic Resonance (NMR) spectroscopic investigations and DFT calculations, mainly of NMR parameters. Both the liquid and the solid states are discussed here. This text is a mix of published results supplemented with new findings. This paper deals with examples in which useful results could not have been obtained without combining NMR measurements and DFT calculations. Examples of such cases are tautomeric systems in which NMR data are calculated for the tautomers; hydrogen-bonded systems in which better XH bond lengths can be determined; cage compounds for which assignment cannot be made based on NMR data alone; revison of already published structures; ionic compounds for which reference data are not available; assignment of solid-state spectra and crystal forms; and the creation of libraries for biological molecules. In addition to these literature cases, a revision of a cage structure and substituent effects on pyrroles is also discussed.

## 1. Introduction

**Figure 1:**
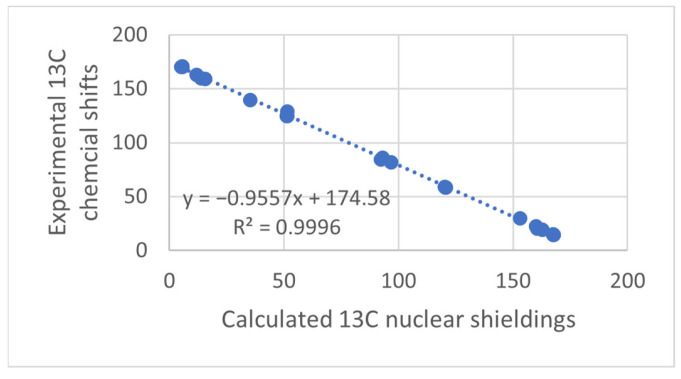
Plot of calculated vs. experimental ^13^C chemical shifts for ethyl (Z)-R-3-(amino)but-2-enoate (see Figure 2). B3LYP is the functional and 6-311 ++ G(d,p) is the basis set. A similar plot is found in Ref. [7].

The NMR technique has reached maturity in many ways, and so have Density Functional Theory (DFT) calculations. Unfortunately, no golden rule about a functional or basis set can be given, so inspiration about the choice of functional and basis set can be found in the papers discussed here. Papers finding the optimal DFT functional and basis set can also be found elsewhere [1,2,3]. Some authors use calculated values for TMS as a reference; this should be avoided, as the correlation between experimental and clouted values is seldom one (see Figure 1). This paper will focus on the synergy obtained by combining these two methods. The examples will be NMR parameters calculated for non-existing structures to be used in tautomeric equilibria, assignment of stereoisomers, assignment of cage compounds, structural studies of charged species, accurate structures of hydrogen bonded systems, a revision of already published structures of natural products [4], creating libraries for biological molecules, and the assignment of solid-state NMR spectra. The present paper covers primarily recent work. Numerous reviews dealing with DFT calculations and NMR at some point are available [5,6].

## 2. Results and Discussion

### 2.1. DFT Calculations

When the text refers to DFT calculations, this normally involves both structure optimization and calculation of nuclear shielding using the Gage Independent Atomic Orbital (GIAO) method [8,9]. For an example, see Figure 1. The same applies to solvent effects if mentioned, e.g., through a Polarizable Continuum Model (PCM) approximation [10].

Reviews on the calculation of nuclear shielding are available [11,12]. An overview of considerations related to DFT calculations is given in Ref. [13]. Their use in natural products has also been demonstrated [14,15,16].

### 2.2. XH Positions

Determining the position of OH or NH protons in hydrogen-bonded systems based on X-ray measurements is often difficult. This is improved by optimizing X-ray structures using ^1^H chemical shifts as constraints in DFT calculations and by taking advantage of the great sensitivity of ^1^H-NMR shielding to hydrogen-bonding properties [17]. The positon of OH protons has also been addressed as a function of solvent. [18]

X-ray structures of proteins are not accurate with respect to the position of NH protons. This is a problem when calculating deuterium isotope effects on ^15^N chemical shifts. This can be improved by performing DFT calculations. In the case of ubiquitin, this was performed by introducing formamide as a hydrogen bond acceptor, with the position of amide oxygen being the same as position of the acceptor in the protein, and then by optimizing the structure using BPW91/6-31G(d,p) calculations [19].

### 2.3. Assignments

Despite the power of 2D NMR, NMR spectra can be so complex that an assignment can be difficult. This has been demonstrated in a large number of natural products [4,20].

**Figure 3:**
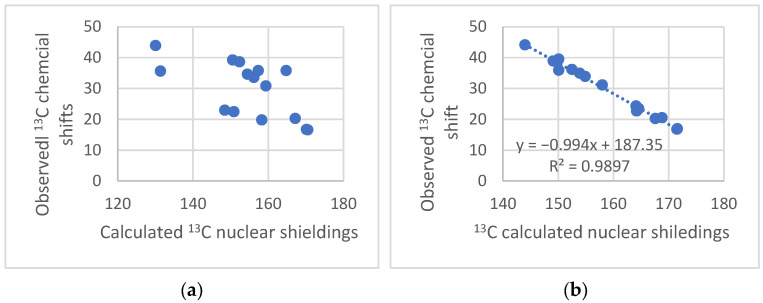
(**a**) Plot of the observed chemical shifts for Hortoishwarane (Figure 4b) [21] vs. calculated nuclear shielding (B3LYP/6-31G(d)). (**b**) Reassigned observed chemical shifts for Hortoishwarane vs. calculated nuclear shielding for (−)-Ishwarane (Figure 4b).

**Figure 4:**
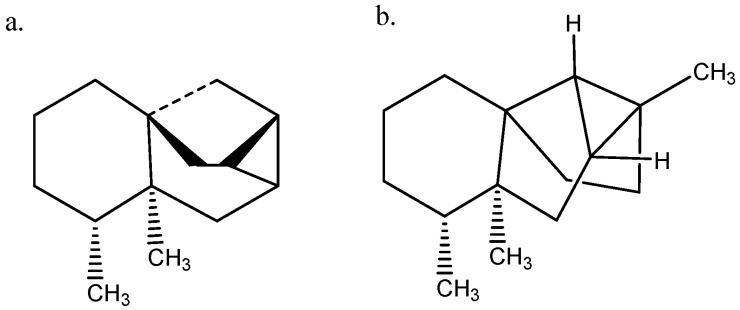
(**a**) (−)-Ishwarane. (**b**) (−)-Hortoishwarane.

An example is given below. An attempt to determine the absolute structure of a molecule isolated from *H. angustifolia* by ^13^C NMR shows that a comparison of experimental and calculated nuclear shielding by Density Functional Theory (DFT) calculations (GIAO approach) gives a very poor fit (Figure 3a), indicating that the assigned structure probably needs revision. This also suggests a reassignment of the ^13^C data. However, the experimentally reassigned ^13^C chemical shifts are very close to those of (−)-ishwarane (Figure 4a). Plotting those new experimental data vs. calculated nuclear shielding gives a very good correlation, which leads to the conclusion that the compound isolated from *H. angustifolia* is (−)-ishwarane (Figure 4a).

### 2.4. Stereochemistry

**Figure 5:**
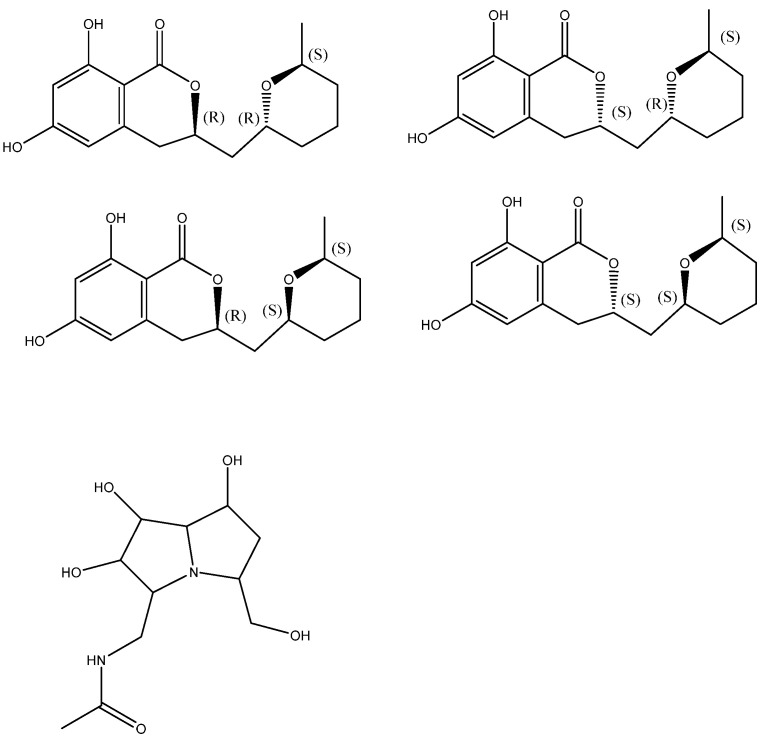
Top four structures: stereoisomers of cladosporin. Bottom: pochonicine. Taken from Ref. [22].

The stereochemical assignment of cladosporin was demonstrated. It has three stereocenters featuring 3*R*, 10*R*, and 14*S* absolute configuration (see Figure 5). The four cladologs shown in Figure 5 were calculated and ^13^C chemical shifts were measured. It was fond that chemical shift differences between pairs of carbons gave better results and the parameter Mean Average Error, MAE_ΔΔδ_, was used for the evaluation of the six sets of data comparing the calculated and experimental data. MAE_ΔΔδ_ = Σ(ΔΔδ)/n_ΔΔδ_. Σ is the summation of n computed Δδ absolute error values (ΔΔδ) normalized to the number of ΔΔδ errors considered (n_ΔΔδ_) [22]. The method was also tested successfully on pochonicine (Figure 5 below) with four chiral centers.

All ^13^C NMR chemical shift data were assigned to each specific isomer [23].

### 2.5. Isotope Effects

**Figure 6:**
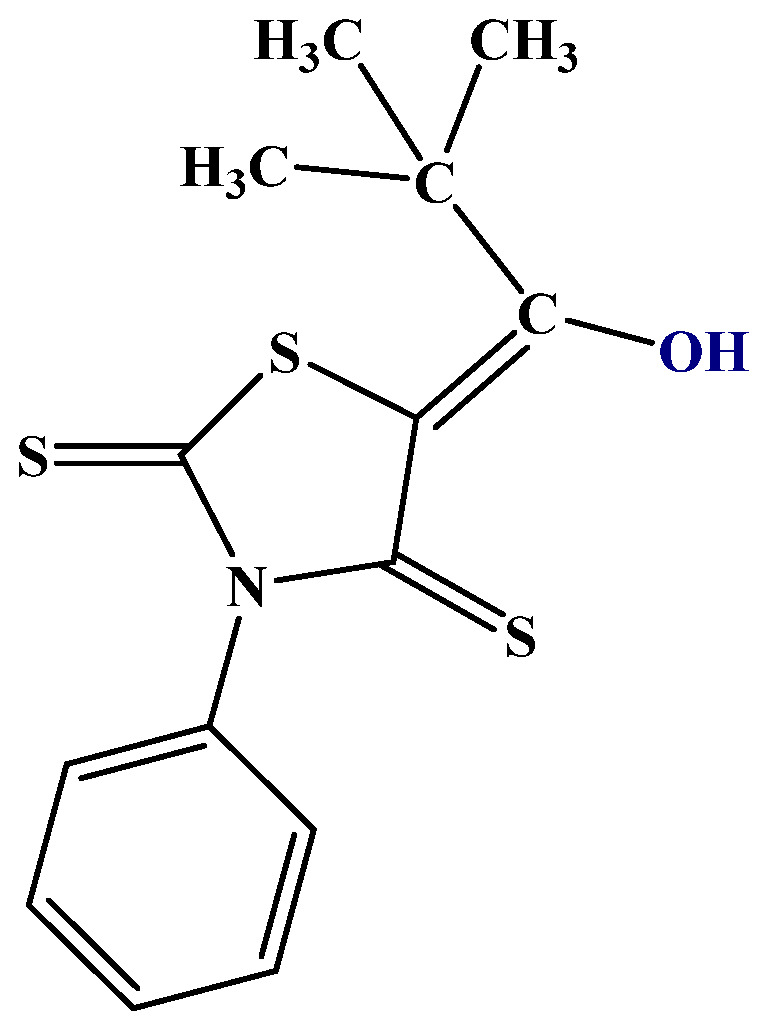
5-tert-butyl-3-phenyl-4-thiorhodanine. Other investigated thiorhodanines with bulky substituents are 5-adamantoyl-3-methyl-4-thiorhodanine and 5-adamantoyl-3-phenyl-4-thiorhodanine [27].

In the study of hydrogen bonding and tautomerism, a useful NMR parameter are isotope effects on chemical shifts. Especially, deuterium isotope effects on ^13^C, ^15^N, and ^19^F chemical shifts have been studied [24]. These can be calculated using the Jameson approach [25]. In its most simple form, deuterium isotope effects can be calculated by assuming that the XH bond is shortened upon deuteration. Assuming a reasonable value and that all the isotope effects of the molecule depend on the same shortening, a plot vs. experimental values can be used to scale the shortening. Isotope effects have been demonstrated in 5-acylrhodanines and the corresponding thiorhodanines. Structure and isotope effects have been calculated using the B3LYP-GD3BJ functional and the 6-311++G(d,p) basis set. Based on these calculations, the compounds were shown to exist in the enol form (Figure 6) rather than the thiol form or the keto-thione form [26]. This is rather unusual as β-thioxoketones have been reported to be tautomeric [24].

**Figure 7:**
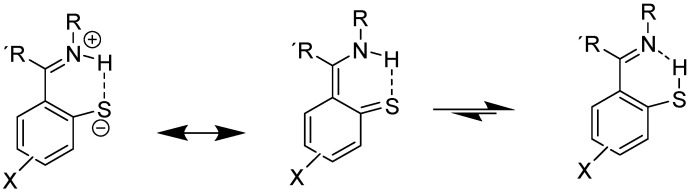
Resonance and tautomeric forms of thiophenoxyketimines. R alkyl or aryl, R methyl.

An example involving NH and C=S bonds is thiophenoxyketimines (Figure 7). In this case, the best DFT functional is B3LYP/6-311+G(2d,p), including solvent effects. The chemical shift for the C=S carbon is very low, 166 ppm, showing a large contribution of the zwitter ionic form (Figure 7). A small percentage of the SH form is possible based on the calculated ^13^C chemical shifts [27].

### 2.6. Tautomerism

**Figure 8:**
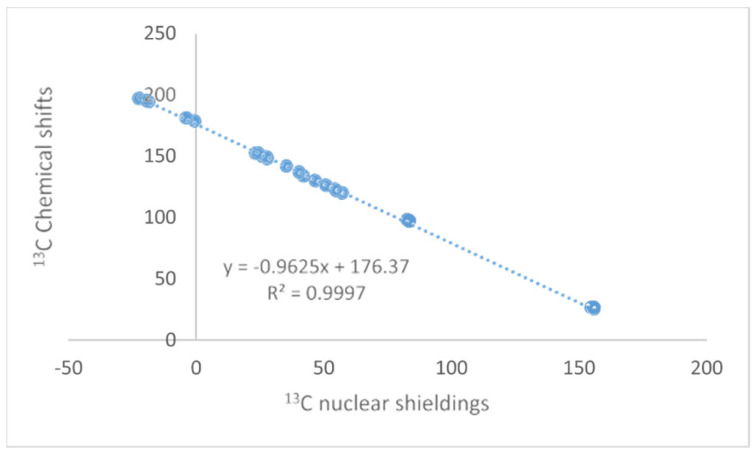
Plot of experimental ^13^C chemical shifts vs. calculated ^13^C nuclear shieldings keto-enol forms of 1-(n-pyridinyl)butane-1,3-diones. Taken from Ref. [28]. Copyright Wiley 2023.

DFT calculations can provide structural information on and energies of the two tautomers, and also, in this context, nuclear shieldings of the nuclei. Knowing the latter, equilibrium constants can be determined. Examples are many, including β-diketones, β-thioxoketones, *o*-hydroxySchiff bases, or *o*-hydroxyazo compounds [24]. One case is shown in Figure 8 for 1-(n-pyridinyl)butane-1,3-diones. The calculated nuclear shieldings (B3LYP/6-311++G(d,p)) of both structure and nuclear shielding calculations for the two tautomers are weighted according to their calculated energies [28].

**Figure 9:**
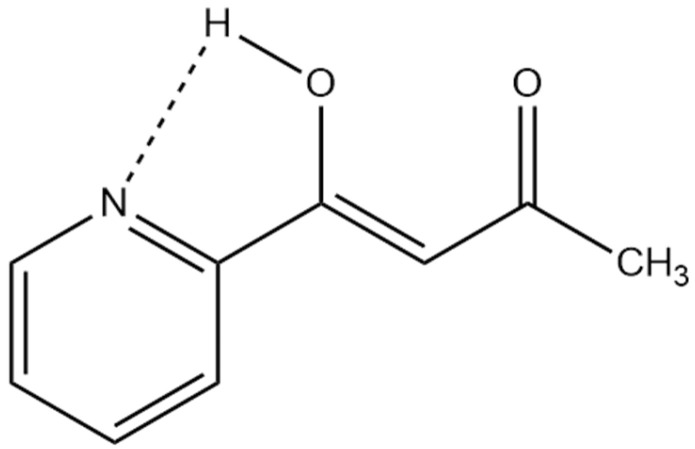
Possible hydrogen bond in 1-(n-pyridinyl)butane-1,3-dione other than the strong one to oxygen [28].

In cases in which other intramolecular effects, such as hydrogen bonding to nitrogen, is present, DFT calculations of the structure are invaluable (see Figure 9).

**Figure 10:**
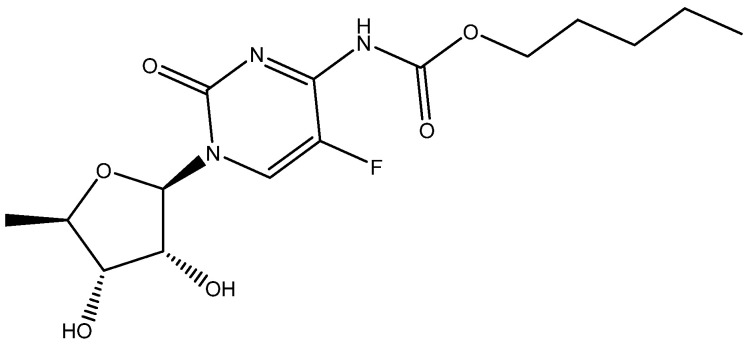
One of the tautomeric structures of capecitabene.

Cmoch et al. [29] investigated the structure of capecitane (see Figure 10) in various solvents and used DFT calculations to produce chemical shifts which could be observed not unambiguously.

**Figure 11:**
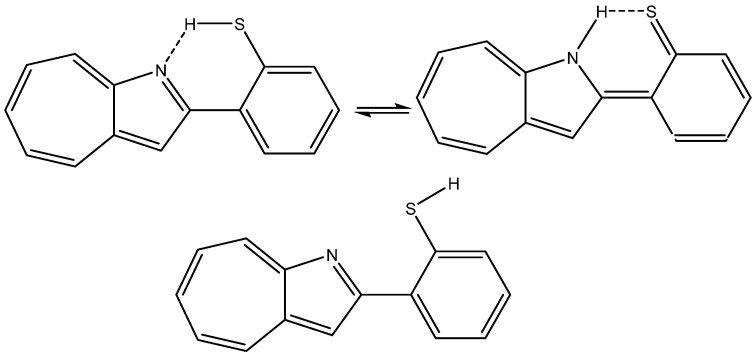
Tautomers and rotamers of 2-(2-mercaptophenyl)-1-azaazulene from Ref. [30].

A case in which the calculated nuclear shielding of both hydrogen bonds and non-hydrogen bonds (XH, X=N or S) have been calculated but not used is 2-(2-Mercaptophenyl)-1-azaazulene (see Figure 11), despite the fact that the large spread in ^1^H chemical shifts (from ~18 to ~3 ppm) offers a platform for analysis [30].

**Figure 12:**
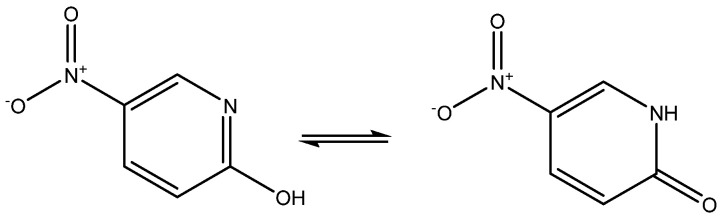
Tautomers of 2-hydroxy-5-nitropyridine.

2-Hydroxy-5-nitropyridine may exist as two keto-eno tautomers (see Figure 12). Its NMR parameters were calculated and it was found that they fitted best with the keto-form [31], although the NMR parameter possibly should have been averaged, as the energy difference between the two tautomers was as low as 1.35 Kcal/mol.

**Figure 13:**
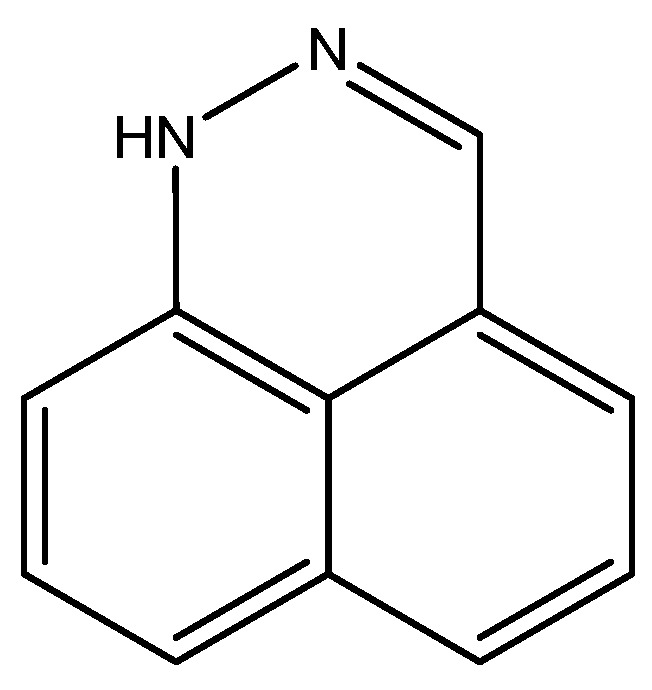
Structure of benzo[d,e]cinnoline.

For benzo[d,e]cinnoline, a good fit was found between ^1^H calculated chemical shifts (B3LYP/6-311++G(d,p) for both structure and for GIAO calculations) and experimental values for the tautomer, which are shown in Figure 13. Notice that many other tautomers are possible. The calculated ^13^C chemical shifts form a good base for further studies [32].

**Figure 14:**
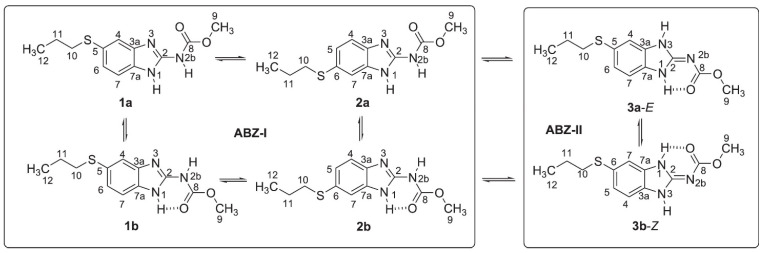
Tautomers and rotamers of albendazole. Taken from Ref. [33]. Copyright Elsevier 2022.

Albendazole may exist both as tautomers and rotamers, as shown in Figure 14 [33]. As seen, the interplay of a very complex set of structures may occur. DFT calculations turn out to be absolutely essential.

Mebendazole is very similar to albendazole. The former is missing the SCH_2_CH_2_CH_3_ side chain. Mebendazole may exist as three different phases related to tautomers [34] and can be defined as desmotropes, i.e., crystal phases in which different tautomers are isolated [35].

**Figure 15:**
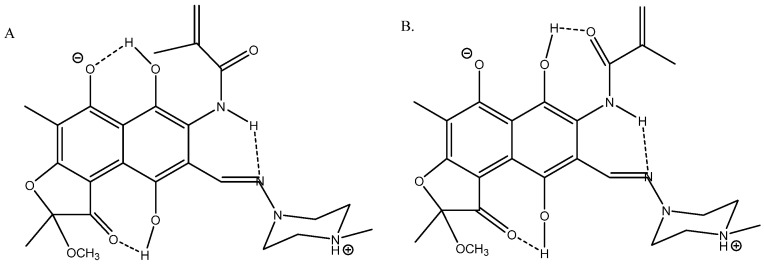

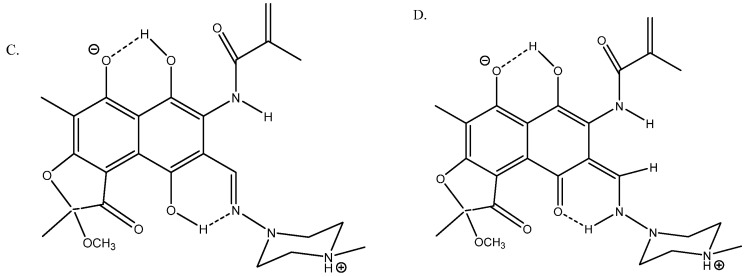
Truncated structures of rifamycin. (**A**) Hydrogen bonds to O^-^ and C=O. (**B**) Hydrogen bonds to C=O(NH) and to C=O. (**C**) Hydrogen bonds to O^-^ and to C=N. (**D**) Is a tautomer of C. Taken from Ref. [36]. Copyright MDPI 2023.

Rifamycin is a complex multi-hydrogen-bonded system [36] (see Figure 15). Its geometries were optimized using the B3LYP functional with the Pople basis set 6-31G(d) [37] and the solvent (DMSO) was taken into account in the PCM approach in the calculations of ^13^C chemical shifts. A very good fit was obtained between calculated and experimental ^13^C chemical shifts; R^2^ = 0.9949 for structure A.

**Figure 16:**
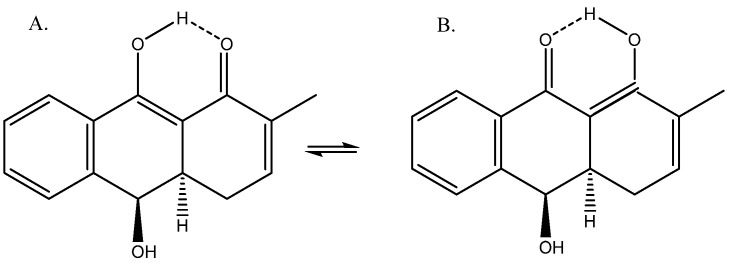
(**A**) Enolic forms. (**B**) Tautomeric structures of dihydroanthracene-1(4H)one.

A compound isolated from Rubia phillipinensis [38] showed a β-diketone-like structure (Figure 16). The author only suggested measurements for structure A based on low temperature, but as the barrier was low, this was not conclusive evidence. DFT calculations of the ^13^C chemical shifts suggested a tautomeric equilibrium between the two forms, with A dominating (9:1) [39].

**Figure 17:**
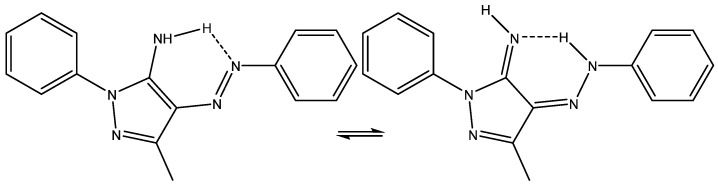
Tautomerism of 3-methyl-1-phenyl-4-(phenyldiazenyl)-1H-pyrazol-5-amine.

The tautomerism of 3-methyl-1-phenyl-4-(phenyldiazenyl)-1H-pyrazol-5-amine (see Figure 17) was studied in detail by calculating ^1^H and ^13^C chemical shifts and comparing them to the experimental results in order to find the best functional and basis set. However, it is strange to see the chemical shift of the methyl group being taken into account, as this is very, very far from the remaining data points [40].

**Figure 18:**
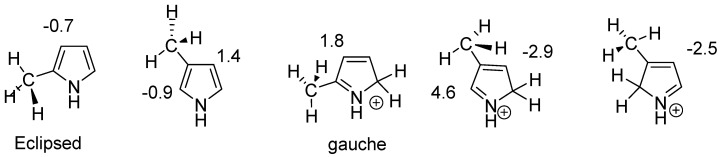
Calculated substituent effects on ^13^C chemical shifts for protonated alkyl pyrroles.

Conformers clearly influence chemical shifts but often are not available directly if the barrier to interconversion is low. Conformers are discussed in Ref. [30]. An example of conformers of protonated alkylpyrroles is given in Figure 18. Different substituent effects are found over the same bond. These substituent effects are later used to estimate the effects of protonation of dimers and trimers (see Section 2.10).

### 2.7. Geometric Isomers

Chemical shifts in the geometric isomers of 18:3 conjugated linolenic acids (CLnAs), hexadecatrienyl pheromones, and model triene-containing compounds were calculated using functionals such as B3LYP and PBE0 as well as functionals including corrections for dispersion interactions (B3LYP-D3, APFD, M06-2X and ωB97XD). ^1^H NMR chemical shifts were computed at the GIAO/B3LYP/6-311+G(2d,p) level, including PCM, or with even less demanding functionals and basis sets. The very good linear correlation found between experimental NMR chemical shifts, δ_exp_, and calculated shifts, δ_calc_, provides a strong indication that the assignment procedure is correct. The procedure led to a correction of previous assignments [41].

### 2.8. Coupling Constants

**Figure 19:**
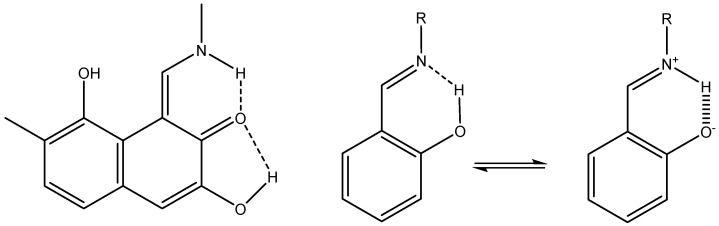
Left: truncated version of gossypol. Right: tautomers of *o*-hydroxyazo compounds.

**Figure 20:**
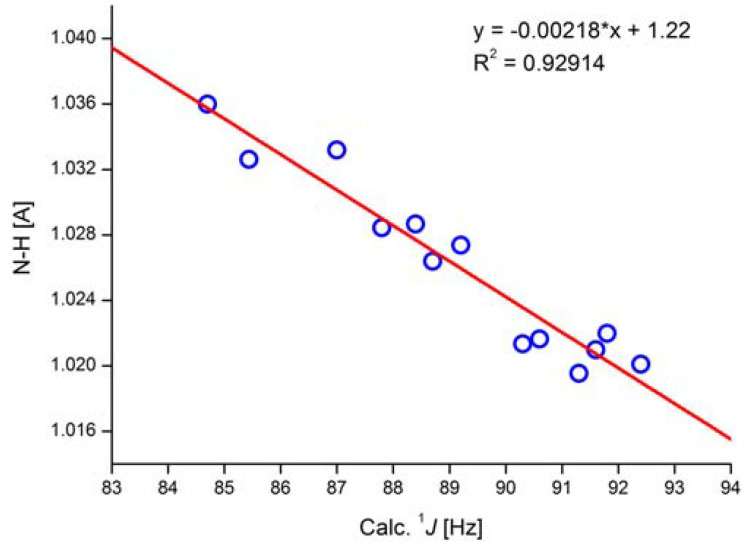
Correlation of calculated NH bond length and calculated one-bond NH coupling constants. In the equation * is a multiplication sign. APFD/6- 311++G** (mixed) functional/basis set. Taken from Ref. [43]. Copyright Wiley 2020.

A parameter often used in systems in which NH groups are central, e.g., tautomerism, is ^1^J(N,H). The study of ^1^J(N,H) has been pioneered by Lycka and his group [42]. ^1^J(N,H) couplings are typically of the order of -90 Hz. However, the magnitude may vary due to substituent effects. A truncated gossypol molecule was chosen as the test system for calculations (Figure 19). It was demonstrated that structure optimization using B3LYP/6-311++G** in PCM approximation (CDCl_3_ as solvent) and APFD/6-311++G** (mixed) for the calculation of coupling constants gave the best result. Studying a number of compounds, a correlation between ^1^J(N,H) and NH bond length was found (see Figure 20) [43].

Taking the equation in Figure 20, coupling constants can be predicted for the NH tautomer of *o*-hydroxyazo compounds (Figure 19). More importantly, it could also be determined that the experimental coupling constant of 45 Hz must come from a tautomeric equilibrium and not from a stretch of the NH bond [43].

**Figure 21:**
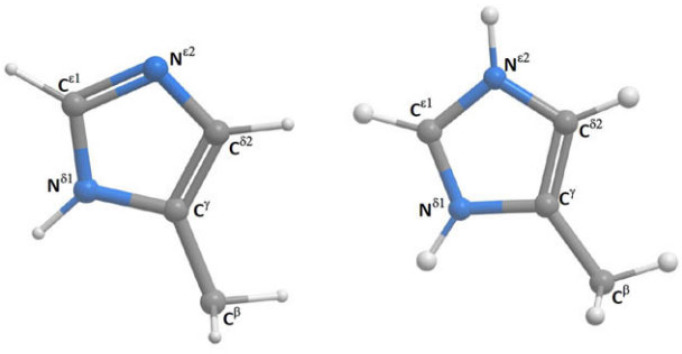
Histidine and protonated histidine. Taken from Ref. [44]. Copyright Elsevier 2017.

Histidine may at high pH exist as two different tautomers (see Figure 21). DFT calculations are used to determine the ^1^J(C,H) couplings. ^1^J(CεH) is not very sensitive to the position of hydrogen, whereas ^1^J(Cδ2H) couplings show a difference of ~15Hz between the two tautomers and are a good probe to identify the tautomeric composition of a non-protonated histine [44].

**Figure 22:**
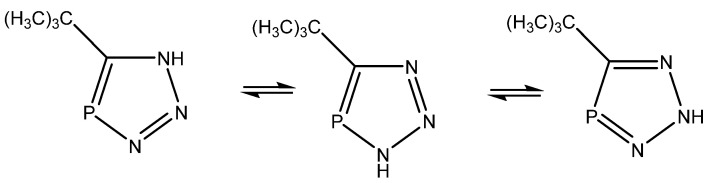
Tautomeric equilibrium of triazaphospholes.

Alkorta and Elguero [45] studied triazaphospholes (Figure 22) and found that the calculated coupling constants (B3LYP/6-311++G(d,p)) in general fitted the experimental ones, except for ^1^J(C,P), and pointed towards the tautomer to the right, whereas the calculated chemical shfits pointed towards the middle one. The authors leave the presence of both tautomers as a possibility. Synergy was not very obvious in the present case.

Bifulco et al. [46] calculated ^13^C-^13^C one-bond coupling constants of the 12 possible stereoisomers of strychnine and assigned the correct stereoisomer by comparison with the experimental values. The functional/basis set was B3LYP/6-311+G(d,p).

### 2.9. Ring Current and Anisotropy Effects

XH (X=O,N,S) chemical shifts are often used as an indicator of hydrogen bonding and as a measure of hydrogen bond strength. However, in aromatic systems, ring currents may influence chemical shifts. A modern way of estimating those is through space NMR shieldings (TSNMRS) [47].

**Figure 23:**
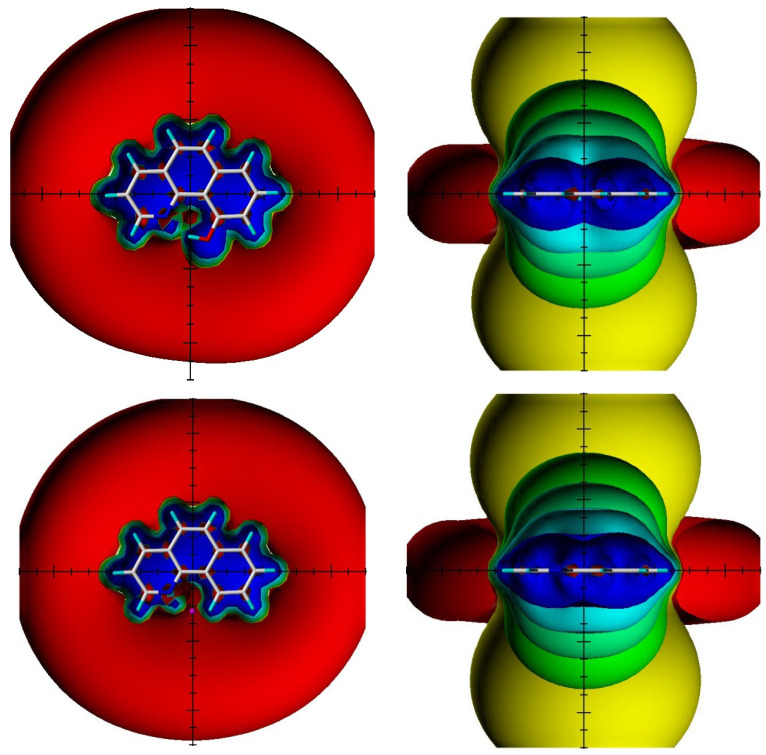
Visualization of the spatial magnetic properties (TSNMRS) of 10-hydroxybenzo[*h*]quinoline (**above**) and benzo[*h*]quinoline (**below**) as ICSS of different direction and size (blue represents 5 ppm shielding, cyan 2 ppm shielding, green-blue 1 ppm shielding, green 0.5 ppm shielding, yellow 0.1 ppm shielding, and red −0.1 ppm deshielding). The pink dot in the top view of benzo[*h*]quinoline shows the position of the OH atom in 10-hydroxybenzo[*h*]quinolone. Taken from Ref. [48]. Copyright Elsevier 2018.

An example is shown in Figure 23 for 10-hydroxybenzo[*h*]quinolone [48].

TSNMRS can also be used to estimate anisotropy effects [49]. TSNMRS values are a combination of ring current effects and anisotropy effects. The TSNMRS value for 10-hydroxybenzo[*h*]quinoline is as high as −1.91 ppm. For 1,3,5-triacetyl-2,4,6-trihydroxybenzene, this value is 0.17 ppm; for salicylaldehyde, it is −0.67 ppm; for thiosalicylaldehyde, −0.03 ppm; for the enol form of acetylacetone, 0.40 ppm; for *o*-hydroxyacetophenone, −0.65 ppm; and for *o*-hydroxythioacetophenone, 0.08 ppm [48]. It is interesting to notice the very large difference between salicylaldehyde and thiosalicylaldehyde as well as between *o*-hydroxyacetophenone and *o*-hydroxythioacetophenone. This illustrates the effects of anisotropy. Kleinpeter et al. [50] used TSNMRS to estimate the anisotropy effects of three-membered rings.

### 2.10. Charged Species

NMR of charged species has recently been reviewed [51,52]. Standard chemical shifts of charged molecules are few. DFT calculations of chemical shifts can be extremely useful in structure elucidation. This is demonstrated in the product of acid dimerization of 2,4-dimethyl-3-ethylpyrrole (kryptopyrrole). The best functional and basis set turned out to be B3LYP/6-311++G(2d,p) including PCM. Obviously, the effect of counter ions should be considered [53].

Working on a set of monoprotonated alkylpyrroles, a large set of functionals and basis sets were tested [54].

**Figure 24:**
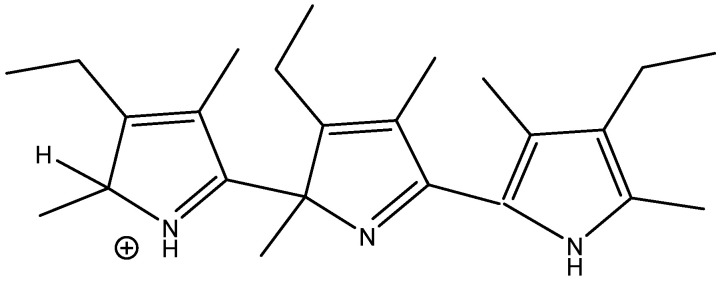
Trimer from acid treatment of krytopyrrole after long standing.

**Figure 25:**
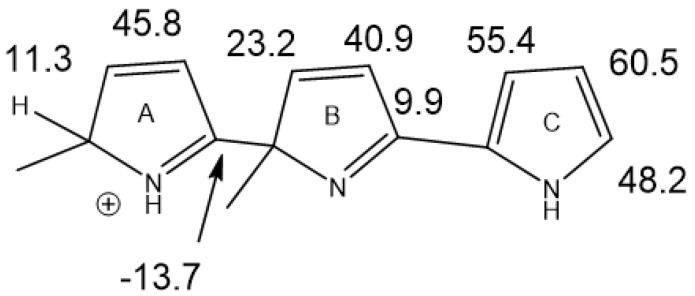
Calculated ^13^C nuclear shielding for a trimer scaffold. The structure was optimized with the functional/basis set B3LYP/cc-pVDZ and the nuclear shielding calculated as B3LYP/6-311++G(2d,p).

The treatment of alkylpyrroles, e.g., kryptopyrrole, may also lead to trimers. Leaving the reaction mixture for a while leads only to one species, as shown in Figure 24. Chemical shifts were calculated based on the values of a scaffold (Figure 25) and substituent effects were calculated based on monomers. A very decent fit between the experimental and calculated values (R^2^ = 0.984) was obtained. It is important to realize that monomers corresponding to rings A or B cannot be synthesized. The calculated results also revealed that only ring A carried a positive charge (notice the large difference in chemical shifts between the charged and the neutral ring). This method can successfully be used for polymers.

**Figure 26:**
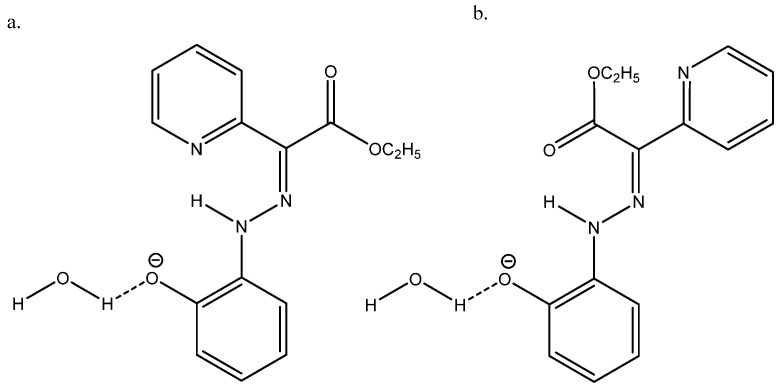
(**a**) is the major form and (**b**) is the minor form. Taken from Ref. [56]. Copyright Wiley 2021.

Nuclear shieldings can be difficult to calculate in negatively charged species due to large self-interaction errors [55]. In the case of the azo compound ethyl 2-(2-(2-hydroxyphenyl)hydrazineylidine)-2-(pyridin-2-yl)acetate (see Figure 26), the best agreement between the experimental ^13^C chemical shifts and calculated ones was obtained with B3LYP/6-311++G(2d,p) + PCM and adding a water molecule, as seen in Figure 26 [56].

### 2.11. Chemical Libraries

Investigating complex biological samples requires access to NMR spectra of a very large number of compounds. For some compounds, reference data are not available. This has been overcome by creating a database of DFT calculated spectra using a method called ISCLE (In Silico Chemical Library Engine) [57].

### 2.12. Solid State

The assignment of solid-state (SS) spectra is often difficult due to overlap. This can be helped by periodic DFT calculations. This is demonstrated in perovskites [58]. Blanc et al. [59] used DFT calculations for a series of silicon catalysts [(≡SiO)M(ER)(=CH*t*Bu)(R′)] (M = Re, Ta, Mo or W; ER = C*t*Bu, NAr or CH_2_*t*Bu; R′ = CH_2_*t*Bu, NPh_2_, NC_4_H_4_) on an oxide support to integrate static values for Chemical Shift Anisotropy (CSA) values. A comparison with experimental data showed motional averaging on the NMR time scale. The data also showed that the motion is non-isotropic.

**Figure 27:**
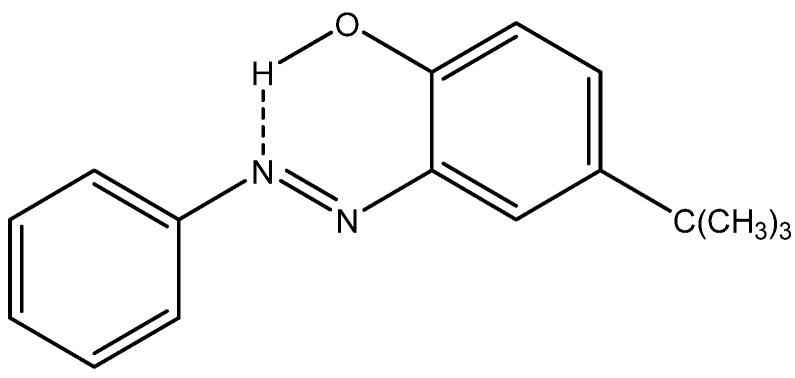
*o*-Hydroxyazo compound on the azo form.

A series of functionalized (NH or OH) azo-compounds (for an example, see Figure 27) has been studied in the solid state by indirect detection of ^14^N. CASTEP calculations [60] can be corrected by using a hydride PBE0 functional to give better nuclear shieldings:

s(crystal, PBE0) = s(crystal, PBE) + s(gas PBE0)

The tautomeric form can thus be determined. The authors find that “even when the crystal structure is not known, a comparison of gas-phase calculated ^13^C chemical shifts of both possible tautomeric structures with those obtained experimentally by SS-NMR can be used for the determination of the tautomeric form” [61].

The examples above (Figure 27) determine one of the tautomeric forms. It becomes more complex when a tautomeric equilibrium is possible between two species.

^1^H chemical shifts in six solid amino acids are determined and calculated at the DFT and coupled-cluster levels, including spin-orbit coupling, molecular dynamics, and nuclear quantum effects. Including these effects leads to a more accurate prediction of ^1^H chemical shifts [62].

## 3. Conclusions

NMR is a very strong tool in structural studies and a very large amount of data are now available. Where this is not the case, DFT calculations of both structures and NMR parameters can be of great importance. This paper illustrates this in cases like tautomerism, in which the necessary structures and NMR parameters of non-isolable tautomers can be calculated. The same is true for ionic species or for compounds found in biological samples. A revision of these structures is also demonstrated. A new case is that of hortoishware. It is also demonstrated that substituent effects on chemical shifts can be calculated for use in polymers. Structural studies of monounsaturated and ω-3 polyunsaturated free fatty acids is discussed in a paper in this Special Issue [63]. The assignment of solid-state spectra and crystal forms also profit greatly from DFT calculations.

## Figures and Tables

**Figure 2:**
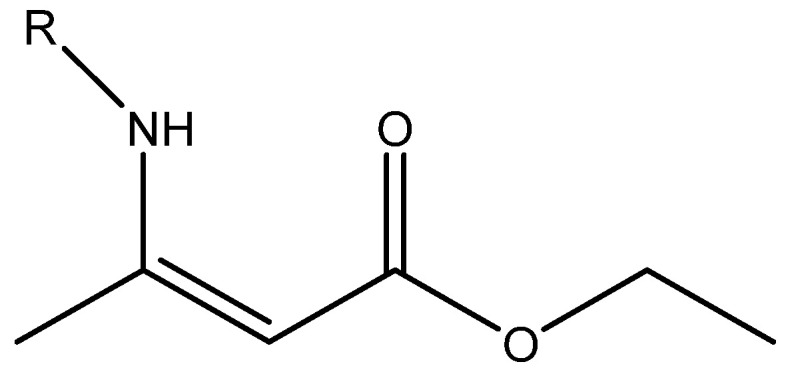
Ethyl (Z)-R-3-(amino)-2-enoate; R being H, Me, Ph, and CH_2_Ph.

## Data Availability

Data are contained within the article.
